# IMPROVEMENTS OF ^111^IN SPECT IMAGES RECONSTRUCTED WITH SPARSELY ACQUIRED PROJECTIONS BY DEEP LEARNING GENERATED SYNTHETIC PROJECTIONS

**DOI:** 10.1093/rpd/ncab056

**Published:** 2021-04-22

**Authors:** T Rydén, W Emma, M Van Essen, J Svensson, P Bernhardt

**Affiliations:** 1 Department of Medical Physics and Bioengineering, Sahlgrenska University Hospital, 413 45 Gothenburg, Sweden; 2 Department of Clinical Physiology, Sahlgrenska University Hospital, 413 45 Gothenburg, Sweden; 3 Department of Oncology, Institution of Clinical Science, Sahlgrenska Academy, University of Gothenburg, 413 45 Gothenburg, Sweden; 4 Department of Medical Radiation Sciences, Institution of Clinical Science, Sahlgrenska Academy, University of Gothenburg, 413 45 Gothenburg, Sweden

## Abstract

The aim was to improve single-photon emission computed tomography (SPECT) quality for sparsely acquired ^111^In projections by adding deep learning generated synthetic intermediate projections (SIPs). *Method:* The recently constructed deep convolutional network for generating synthetic intermediate projections (CUSIP) was used for improving 20 sparsely acquired ^111^In-octreotide SPECTs. Reconstruction was performed with 120 (120P) or 30 (30P) projections, or 120 projections with 90 SIPs generated from 30 projections (30–120SIP). The SPECT reconstructions were performed with attenuation, scatter and collimator response corrections. Postfiltered 30P reconstructed SPECT was also analyzed. Image quality were quantitatively evaluated with root-mean-square error, peak signal-to-noise ratio and structural similarity index metrics. *Result:* The 30–120SIP reconstructed SPECT had statistically significant improved image quality parameters compared to 30P reconstructed SPECT with and without post filtering. The images visual appearance was similar to slightly filtered 120P SPECTs. Thereby, substantial acquisition time reduction with SIPs seems possible without image quality degradation.

## INTRODUCTION

^111^In-octreotide SPECT (single-photon emission computed tomography) has been a cornerstone within nuclear medicine diagnosis of somatostatin receptor–positive neuroendocrine tumors (NETs) and is important for patient selection for ^177^Lu-DOTATATE treatments^(1)^. Recently, ^177^Lu-DOTATATE was approved for treatment of NETs which further has increased the interest of accurate diagnosis of somatostatin receptor–positive NETs. However, ^111^In-octreotide SPECT has the drawback of limited spatial resolution, rather high noise level and long acquisition times. Therefore, ^68^Ga-DOTATOC PET which offers better image quality properties, has increased in attractiveness. However, the short physical half-life of ^68^Ga (*T*_1/2_ = 68 min) limits its use for measurement of late timepoints, which ^111^In, due to its long physical half-life (*T*_1/2_ = 2.7 days), is able to. To our knowledge, it is still not verified which image modality is optimal for patient selection to ^177^Lu-DOTATATE treatment.

The most commonly used SPECT/CT reconstruction method today is the iterative ordered subset expectation maximization (OSEM) algorithm^(2)^. With the OSEM reconstruction attenuation, scatter and collimator-detector resolution corrections (ASCCs) can be performed, which improves image quality^(3)^. Further improvements might be achieved by Monte Carlo (MC)-based OSEM reconstruction methods^(4–6)^. The beneficial value of applying MC techniques for ASCCs it that the three corrections are performed in a united correction when the photons path from decay site in the patient to the gamma camera crystal is simulated. However, no ASCC technique corrects for poor counting statistics. The method for this is to increase acquisition times. For obtaining low noise level in ^111^In-octreotide SPECT acquisition times of about 30 min are required. Clinical protocols often involve 60 frames of 30-s acquisitions. Each frame collects two projections, i.e. totally 120 projections are collected during a 30-min SPECT scan.

The aim of this study was to reduce the SPECT acquisition time by reducing the number of projections, from 120 to 30, and compensate the image quality degradation by including synthetic intermediate projections (SIPs) in the reconstruction. For evaluation, we analyzed quantitatively the image quality of 20 ^111^In-octreotide SPECT images reconstructed with and without SIPs.

## MATERIALS AND METHODS

### Subjects and image acquisition

In the study, 20 ^111^In-octreotide SPECT image data were retrospectively selected. The retrospective use of the image data and waiver of consent were approved by the Regional Ethical Review Board in Gothenburg.

The gamma cameras used for the examinations during this period were Millennium VG Hawkeye, Infinia Hawkeye 4 and Discovery 670 (General Electric Medical Systems, Milwaukee, WI, USA), all with a crystal thickness of 5/8″ and equipped with a medium-energy parallel-hole collimator. For ^111^In-octreotide examinations, a 20% energy window over the 245-keV photon peak was used. The clinical SPECT images were acquired 1-day post injection with 110–220 MBq ^111^In-octreotide with a 30-s frame time duration for 120 projections. The matrix size was 128 × 128 with a pixel size of 4.42 mm and a slice thickness of 4.42 mm. The CT images used in the SPECT/CT reconstructions were acquired using a 140-kV tube voltage, 2.5 mAs and a rotation speed of 2.6 rpm. The matrix size was 512 × 512 with a pixel size of 0.98 mm and a slice thickness of 5 mm.

### The convolutional neural network

In the present study, we used our recently constructed deep neural network: the deep Convolutional U-net–shaped neural network for generation of Synthetic Intermediated Projections (CUSIP)^(7)^. We use three trained CUSIP for generating 90 SIPs from 30 acquired projections. The details of CUSIP were earlier described by Rydén et al.^(7)^ Below follows a short description of the construction of CUSIP.

**Figure 1:**
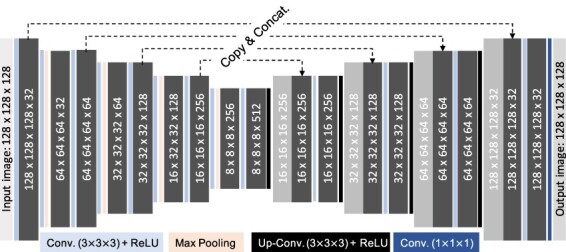
Schematic description of CUSIP with the number of filters indicated in the convolution steps.

CUSIP has a three-dimensional structure and consist of encoder and decoder units with skip connections between the corresponding layers (Figure 1). The input matrix (image) consists of 30 normalized projections with a matrix size of 128 × 128, which is concatenated to a cubic matrix of (128 × 128 × 128). During the development of CUSIP it was concluded that a cubic matrix generated improved results, therefore this arrangement of the input matrix. Thereafter, input image is convolved and down-sampled in the encoder part, which consists of convolutional layers followed by a rectified linear unit (ReLU) activation function. After each down-sampling step, with max pooling, the feature channels are doubled. For up-sampling, the decoder unit used transposed convolutional layers followed by a ReLU activation function. The number of feature channels are halved after each step of up-sampling.

Three different CUSIPs were trained, with 352 ^177^Lu (75%) and ^111^In (25%) images, to yield the following three SIP sets: (1) projections 2, 6, 10…118; (2) projections 3, 7, 11…119 and (3) projections 4, 8, 12…120. These projection sets were cropped from the 128 × 128 × 128 matrix output images (Figure 1). In the present study, these three CUSIPs were used for generating the 30–120SIP data set of 20 diagnostic ^111^In-octreotide SPECT images, down-sampled from 120 to 30 projections.

### SPECT reconstructions

SPECT images for quality evaluation were reconstructed for three sets of projections: (1) the 120 acquired projections (120P), (2) 30 projections using every fourth projection of the 120 (30P) and (3) 120 projections derived from 90 (3 × 30) SIPs generated from the 30 projections using the three CUSIPs (30–120SIP). The SPECT/CT reconstructions were performed with the Sahlgrenska Academy Reconstruction code (SAREC) for generating attenuation, scatter and collimator-detector resolution corrected, ASCC–OSEM, reconstructions^(4)^. The SARec code use MC simulation techniques, and 6 subsets and 10 iterations were used in this study, with a simulation time of 4 min. The 30P ASCC–OSEM were also postfiltered with a Gaussian filter, SD of 4 mm, (ASCC–OSEM 30GF), and a Butterworth filter, order of two and cut-off frequency of 0.036, (ASCC–OSEM 30BW).

### Quantitative image quality evaluation of SPECT images

For evaluation of the image quality of the SIPs and the reconstructed SPECT images three quantitative metrics was used. These were: the root-mean-square error (RMSE), peak signal-to-noise ratio (PSNR) and structural similarity index metrics (SSIM). These metrics estimate the image quality compared with a reference image. In the case with SIPs the reference images are the acquired projections. For the SPECT images 30P and 30–120SIP the reference is the reconstruction of all 120 acquired projections (120P SPECT).

The RMSE is square root of the quadratic mean of differences between image (IM) and reference image (RI):

(1) \begin{equation*} \mathrm{RMSE}=\sqrt{\frac{1}{\mathrm{nml}}{\sum}_x^n\sum_y^m\sum_z^l{\left(\mathrm{IM}\left(x,y,z\right)-\mathrm{RI}\left(x,y,z\right)\right)}^2} \end{equation*}

*n*, *m* and *l* are the number of voxels in each direction of the SPECT image. $\mathrm{IM}\big(x,y,z\big)$ and $\mathrm{RI}\big(x,y,z\big)$ refer to the *x*, *y* and *z* coordinates in the SPECT images. For projection images IM and RI are changed to represent 2D images; $\mathrm{IM}\big(x,y\big)$ and $\mathrm{RI}\big(x,y\big)$.

From the RMSE the PSNR is derived [Equation (2)]. PSNR specifies the ratio of the maximal pixel intensity to the power of distortion compared with the RI:

(2) \begin{equation*} \mathrm{PSNR}=20\ {\log}_{10}\left(\frac{\operatorname{MAX}}{\mathrm{RMSE}}\right) \end{equation*}

where MAX is maximum voxel value in the image. Compared with RMSE the PSNR also include the dynamic range of the image and can be regarded as an approximation of human perception as perceived change in image noise. While a decrease in RMSE indicate improved image quality it is the opposite for PSNR; an increase indicates improved image quality. Improvement image quality was defined as a statistically significant difference, *p* < 0.05, between the metrics.

SSIM can be described as a perception-based measure that considers image degradation as perceived change in structural information^(8)^. The values of SSIM range from 0 to 1 where a higher value indicates higher similarity between the images. SSIM where calculated by 3 × 3 × 3 kernel size as follows:

(3) \begin{equation*} \mathrm{SSIM}\ \left(\mathrm{IM},\mathrm{RI}\right)=\frac{\left(2{\mu}_{\mathrm{IM}}{\mu}_{\mathrm{RI}}+{c}_1\right)\left(2{\sigma}_{\mathrm{IM}\mathrm{RI}}+{c}_2\right)}{\left({\mu}_{\mathrm{IM}}^2+{\mu}_{\mathrm{RI}}^2+{c}_1\right)\left({\sigma}_{\mathrm{IM}}^2+{\sigma}_{\mathrm{RI}}^2+{c}_2\right)} \end{equation*}

where ${\mu}_{\mathrm{IM}}$is the average of IM, ${\mu}_{\mathrm{RI}}$ is the average of RI,${\sigma}_{\mathrm{IM}}^2$ is variance of IM,${\sigma}_{\mathrm{RI}}^2$ is the variance of RI, and ${\sigma}_{\mathrm{IMRI}}$ is covariance of IM and RI. Two variables ${c}_1$ and ${c}_2$ are used to stabilize the division with a weak denominator defined as:

$$ {c}_1={\left({K}_1L\right)}^2,{c}_2={\left({K}_2L\right)}^2 $$

where *L* is the dynamic range of the voxel-values, ${K}_1$ and ${K}_2$ set by default to 0.01 and 0.03, respectively.

### Statistics

For the quantitative evaluation of SPECT image quality, we analyzed the data using the paired Student’s *t*-test, and applied corrections for multiple testing. The statistical tests were performed in MATLAB (MathWorks, Torrance, California, USA). A *p*-value < 0.05 was considered to indicate statistical significance.

## RESULTS

**Figure 2:**
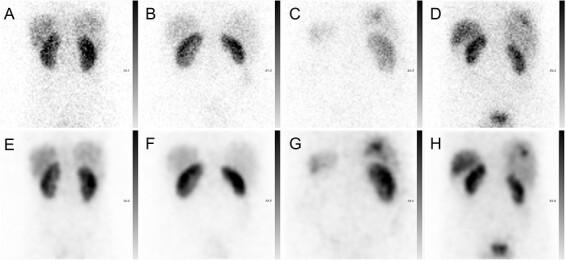
Representative ^111^In-octreotide projections from four patients. The first row presents the patients’ acquired projections (**A**–**D**), whereas the second row presents the generated SIPs for each patient (**E**–**H**).

The 30-s acquired ^111^In projections had rather high noise levels as presented for four representative patients in Figure 2. In contrast, the synthetic projections generated with CUSIP had lower noise levels and a slightly smoother visual appearance compared with acquired projections (Figure 2). The bias, i.e. the mean pixel difference between the acquired and synthetic projections, was close to zero, and both negative and positive differences were present (data not shown).

The image quality parameters are presented in Table 1. The mean RMSE was 1.42, the PSNR was equal to 35.0 dB, and the SSIM was 0.867, indicating high structural similarity between the acquired projections and the SIPs.

**Table 1:** The image quality metrics RMSE, PSNR and SSIM for the SIPs and SPECT images; mean value (standard deviation) for the 20 patients.

Images	RMSE	PSNR	SSIM
SIPs	1.42 (0.32)	35.0 (2.2)	0.867 (0.044)
ASCC–OSEM 30	545 (162)^*^	46.5 (2.4)^*^	0.988 (0.005)^*^
ASCC–OSEM 30–120SIP	387 (119)	48.3 (2.7)	0.993 (0.003)
ASCC–OSEM 30GF	483 (196^*^)^+^	46.5 (2.1)^*^	0.992 (0.004^*^)^+^
ASCC–OSEM 30BW	505 (218)^*^	46.2 (2.0)^*^	0.992 (0.004^*^)^+^

^*^ *p* < 0.001, all tree methods compared with ASCC–OSEM 30–120SIP.

^+^ *p* < 0.05 comparison with and without post filtering of ASCC–OSEM 30P.

**Figure 3:**
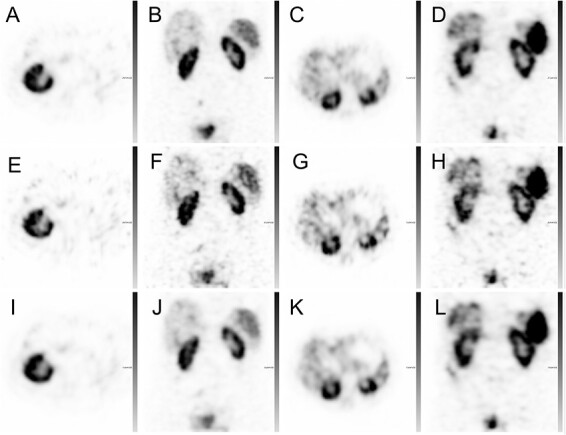
Representative ^111^In-octreotide SPECT reconstructions from four patients: 120P ASCC–SPECT (**A**–**D**), 30P ASCC–SPECT (**E**–**H**) and 30–120SIP ASCC–SPECT (**I**–**L**).

For four out of the 20 patients, the obtained ^111^In SPECT image quality with 30, 30–120SIP and 120 projection sets are demonstrated in Figure 3. With 30 projections a high noise level was visually observed in the SPECT reconstructions. The noise level was decreased with an increased number of projections, either with the 30–120SIP or the 120P sets, and a smoother activity distribution was observed. The reconstruction with the different projection sets resulted in higher deviation in voxel values between the 30P and 120P sets as compared with the deviation in voxel values between the 30–120SIP and 120 projection sets (data not shown). Subsequently, the image quality parameters, RMSE, PSNR and SSIM, were statistically significantly improved between the 30 and 30–120SIP sets (Table 1). Furthermore, the 30–120P had also statistically significant higher RMSE, PSNR and SSIM than the post filtered 30P SPECTs (*p* < 0.001). The post filtering improves SSIM, and with Gauss filtering the RMSE was also improved, which was not the case with Butterworth filtering. None of the post filtering methods improved PSNR. Instead, post filtering had a tendency to decrease PSNR, while not statistically significant.

## DISCUSSION

Our results demonstrate that generation of synthetic ^111^In projections based on a limited set of projections and added to the reconstruction with sparsely acquired projections clearly can improve SPECT image quality. It was especially observed that the apparent noisy character of SPECT reconstruction with sparsely acquired projections was less apparent with the addition of SIPs. Consequently, the proposed method with SIPs should have potential to reduce SPECT acquisition times 4-fold. A reduction SPECT acquisition time from 30 to 7.5 min will have several impacts in clinical routine, such as higher patient comfort and important time saving. The method will still require CT investigation, but this acquisition time is short in comparison to SPECT acquisitions. Further, with reduced acquisition time it can be assumed that image artefacts from patient movement can be reduced. These image artefacts might be minor and not easily detected but have impact on image quality. Larger patient movements during acquisition should be detected by the operator and in these cases a repeated investigation is often required. Reducing these factors are beneficial for economical health costs. However, it should not be on the expense on reduced precision in correct diagnostic decisions which does not seem to be the case with SIPs, although it requires further studies.

Another application of SIPs might be absorbed dose reduction to patients. Diagnostic investigation with ^111^In-octreotide yields rather high effective doses; around 6 mSv^(9)^. By decreasing the injected activity 4-fold and increasing the acquisition time 4-fold in the 30-projection protocol, the 30–120SIP reconstruction protocol could be applied for generating similar image quality as demonstrated in this study but with a 4-fold reduced effective dose. However, this application of SIPs has to be verified in a subsequent study.

In this study we analyzed quantitative image quality measures, i.e. RMSE, PSNR and SSIM. All these measures demonstrate statistically significant improvements when SIPs were added to the sparse acquired projections. The improvements were in the same range as we previously demonstrate for ^177^Lu. Although the 30–120P SPECT images also had a pleasant visual appearance these measures are not able to deduce the highest image quality between 120P and 30–120P SPECTs. Accurate qualitative ranking of SPECT images requires experienced nuclear medicine specialists. Qualitative evaluation was not performed is this study, but in our previous study no statistically significant difference in ranking between 120P and 30–120SIP ^177^Lu images was obtained^(7)^. In that study it was also concluded that the 30P ^177^Lu SPECT was too noisy for clinical investigations. Similar results might be expected for the present study, and qualitative evaluation of ^111^In SPECT reconstructions with SIPs will be analyzed in forthcoming studies.

The image quality of the 30P ^111^In SPECT images could be improved with post filtering, however there were limited improvements in the used quantitative measures. The SSIM was improved for both filters but RMSE just improved for the Gauss filter and neither could improve PSNR. Although not proved, the superiority of using SIPs over post filtering might partly be explained by the addition of counts into the reconstruction, which is not the case with post filtering. Other explanations might be in the network architecture and the weight different filters received during training. Nevertheless, it might be interpretated that CUSIP use different, optimized, filters for various image structures. However, interpretation of the networks setting of weights and the explanation of superior ‘filtering’ with CUSIP, compared with post filtering, remain to be analyzed carefully.

The CUSIP has been trained mainly with ^177^Lu-octreotate SPECT images (75%), but a small number of ^111^In-octreotide SPECT images (25%) was added to improve training result^(7)^. With this approach of adding ^111^In-octreotide SPECT images the training loss was decreased, compared with not using ^111^In-octreotide SPECT, and CUSIP could successfully improve ^177^Lu 30P SPECT images. In this study we evaluated CUSIP performance on ^111^In-octreotide 30P SPECT images and obtained similar improvements in the used image quality measures as obtained for ^177^Lu SPECT images, e.g. the RMSE decrease for 30–120SIP ^177^Lu SPECT and 30–120SIP ^111^In SPECT was 26.3 and 29.2%, respectively. These results also indicate that CUSIP might be able to improve SPECT images with other radionuclides such as ^99m^Tc, which has to be explored further.

## CONCLUSION

In summary, the SIPs generated by the recently constructed CUSIP demonstrated high similarity to the acquired projections. In addition, SPECT image quality with sparsely acquired projections is improved by the addition of SIPs into the reconstruction. The SPECT image quality with SIPs was superior to post filtering of SPECT images reconstructed with sparsely acquired projections. Using SIPs into reconstruction indicates that a 4-fold decrease in acquisition time might be possible. This will improve patient comfort during investigations as well as decrease the risk of image artefacts due to patient movements.
